# Lack of Self-Efficacy and Resistance to Innovation Impact on Insufficient Learning Capabilities: Mediating the Role of Demotivation and Moderating the Role of Institutional Culture

**DOI:** 10.3389/fpsyg.2022.923577

**Published:** 2022-07-26

**Authors:** Shao Yan

**Affiliations:** Jiangxi Vocational College of Finance and Economics, Jiujiang, China

**Keywords:** insufficient learning capabilities, demotivation, lack of self-efficacy, resistance to innovation, second language learners

## Abstract

Learning capabilities have been an essential element for the success of second language learners that needs regulators' and researchers' emphasis. Hence, the current research examines the role of lack of self-efficacy and resistance to innovation on the insufficient learning capabilities of second language students in China. The current study also examines the mediating role of demotivation among the linkage of lack of self-efficacy, resistance to innovation, and insufficient learning capabilities and explores the moderating role of institutional culture among demotivation and insufficient learning capabilities. The researchers have applied the questionnaires method to gather the data from selected students and employed the smart-PLS to assess the measurement and structural models. The results revealed that lack of self-efficacy and resistance to innovation has a significant and positive linkage with insufficient learning capabilities. The results also exposed that demotivation significantly mediates among lack of self-efficacy, resistance to innovation, and insufficient learning capabilities. The findings also explored that the institutional culture significantly moderates the linkage between demotivation and insufficient learning capabilities.

## Introduction

Education is the key to success as well as the development and prosperity of any country. The world is enjoying advancement with the passage of every hour which is giving ease to mankind but on the other hand this advancement re also introducing problems for the mankind. In both the case the advancement as well as problems the ultimate solution is the education. Cultural exchange is taking place as the result of globalization (Makarova et al., 2019; Nguyen and Do, 2020). This cultural exchange forces the nations to learn each other languages with the view to better understanding. This learning of another country's language is narrated as a second language. Second language learning plays a vital role in cultural exchange. There is a number of people travel to other nations for the sake of education, jobs, business etc. One of the common barriers faced by them is learning the language of the country where they are going. Similar is the case with the country which welcomes the foreigners for different purposes. It's the concerned country's government's responsibility to provide a better second language learning system to the country for better cultural exchange. The country that failed to pay focus on such topics witnessed insufficient learning capabilities and failed to match the world requirement. Literature also proposed that second language learners' capabilities play a vital role from a different point of view (Jackson and Ruf, 2018; Mamajonova, 2021).

### Education System in China

**Figure 1:**
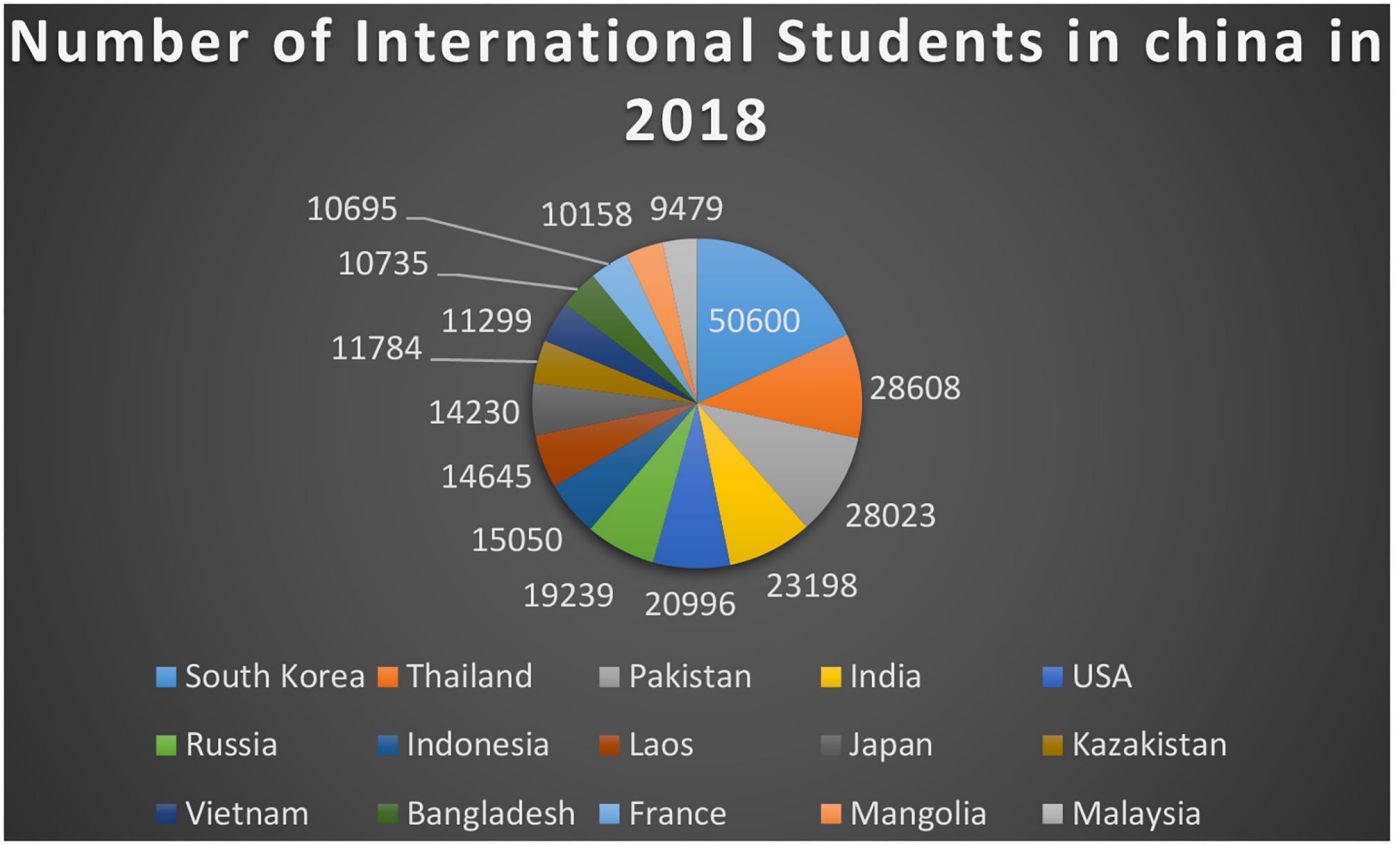
Number of international students enrolled in China.

China is the fastest-growing economy in the world. China is attracting the world for better business as well as educational opportunities. Over the last two decades, there has been an increase in the number of persons learning Chinese as a second/foreign language (CSL/CFL) both inside and outside of China. It has been estimated that by the end of 2020, more than 20 million people from more than 180 countries/regions would be learning Chinese as an extra language. According to a report provided by China's Ministry of Education (MOE), around a quarter million international students were studying Chinese in mainland China in 2019 (MOE, 2019). Despite the drastically expanding number of CSL learners worldwide, the learning process of such learners has been found to be under-researched. A number of pertinent questions require additional investigation, including profiling the repertoire of strategy usage and measuring and enhancing strategic competency in Chinese learning. One of the basic factors which result in the success or failure of the second language learners is the learning strategy. As the importance of learning strategy in second language learning and acquisition has long been recognized (Zhang et al., 2019). The cognitive viewpoint dominated early efforts to learn strategy. This method divides learning strategies into many areas, including learning, communication, and social strategies, meta-cognitive, cognitive, and social-affective strategies, and a six-category learning strategy taxonomy. Literature proposed that the most common issues faced by the second language learners are (1) bored by the traditional learning methods, (2) feeling of embarrassment, (3) timing issues, (4) lack of interaction with a native speaker, (5) institution culture, (6) students response, and (7) introduction of new learning tools like innovation (Mhic Mhathúna and Hayes, 2020; Obeso, 2020; Vivian, 2021; Chien et al., 2021c). Many times these factors result in students' demotivation. Once the learner gets demotivated then it affects ones learning capabilities. The number of international students enrolled in China is given in Figure 1.

### Study Gaps and Contributions

The present study will address some gaps does exist in the literature like (1) being one of the important topics like linguistic along with learning capabilities although researched although but still not reached its peak, (2) Wu et al. (2021), worked on the students learning capabilities development whereas the present study will work on insufficient learning capabilities along with moderation effect of demotivation and moderation effect of institutional culture in China, (3) Hamidova and Ganiyeva (2020), worked on foreign language learning with the view to competence development whereas the present study will work on insufficient learning capabilities along with students demotivation, linguistic knowledge, family support and resistance to innovation in China, (4) Amarakoon et al. (2018), worked on the learning capabilities along with innovation whereas the present study will test the insufficient learning capabilities along moderation and mediation effect in Chinese perspective with a new data set, (5) the present study will check the model in Chinese perspective with new data set, and (6) Ranjha et al. (2021) worked on the demotivation factor in foreign language learning whereas the present study will investigate the insufficient learning capabilities with moderation and mediation effect in China.

The significance of the study are (1) will highlight the importance of second language learning and learners for any country, (2) help professional to revamp their policies for the betterment of second language learning for better performance of the learners as well as country, and (3) will help the researchers to identify and explore the more aspects or issues stands behind the insufficient of learning capabilities, and (4) in China although students begin learning English as a second language on the first day of school. However, the majority of them are unable to properly grasp English as a second language. This research can assist teachers in better understanding the difficulties that students have when learning English. Furthermore, teachers can recognize the effect of a learner's on English learning, and among the faults, they can assist in grammatical correction.

The study structure is divided into five phases. The first phase will present the introduction. In the second phase of the study, the pieces of evidence regarding lack of self-efficacy, resistance to innovation, demotivation, institutional culture, and insufficient learning capabilities will be discussed in the light of past literature. The third phase of the study will shine the spotlight on the methodology employed for the collection of data regarding lack of self-efficacy, resistance to innovation, demotivation, institutional culture, and insufficient learning capabilities and its validity will be analyzed. In the fourth phase, the results of the study will be compared with the pieces of evidence reviewed from the literature. In the last phase, the study implications along with the conclusion and future recommendations will be presented which will conclude the paper.

## Review of the Literature

This section provides the literature of the past studies of understudy constructs. The literature is given under subsections given below:

### Lack of Self-Afficacy and Insufficient Learning Capabilites

For the past few decades, the world is trying to establish and incorporate the elements of self-efficacy among the people. The incorporation of these elements is helpful for the learners as well as for the teachers in the creation of a significant environment for the future. In this context: Stepp and Brown (2021), asserted the lack of a link between self-efficacy and the instruction of teachers and its impact on the capabilities of students. Self-efficacy refers to all elements and factors that describe the essentials for creating abilities among the learners of China. When there are all abilities in respect of self-efficacy there are numerous learning abilities for the learners and teachers. Further, Wang et al. (2021) discussed the roles of self-efficacy and the social services that are important in nursing and the management of learning capabilities. Self-efficacy provides all the essentials in a person that not only helps to achieve the goals but also helps to build all the necessary faiths to counter the challenges. It is prominent that lack of self-efficacy is how damaging to the learning abilities and significantly enumerates that self-efficacy is an attribute to achieve the objective. Additionally, Laruffa (2020) examined the insufficiency of learning capabilities that are based on the enhancement of social policies for self-efficacy. There is a dominant impact of lack of self-efficacy that impacts the insufficient learning abilities and capabilities in China. Finally, Afshari and Hadian Nasab (2021) enumerated the learning capabilities which are not enhanced due to insufficient material and talent that requires proper self-efficacy with intellectual capital. The capabilities are unable to be formed without the preservation and usage of self-efficacy. The cognitive and determined approach is a necessity for sufficient learning capabilities. Lack of self-efficacy refers to the lack of cognitive skills, lack of social experience and lack of observation ability which is an ultimate message of the insufficiency of learning capabilities.

**H1:** Lack of self-efficacy significantly influences insufficient learning capabilities.

**Figure 2:**
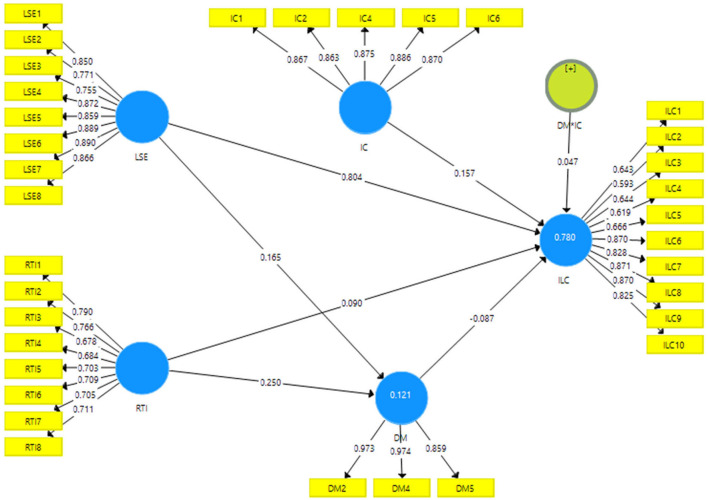
Measurement model assessment.

Figure 2 provided the factor loadings and results indicated that the values are higher than 0.50 and exposed valid content validity.

### Resistance to Innovation and Insufficient Learning Capabilites

The barriers to the rise of freedom as well as the growth of the country refer to the resistance to the innovative approach whether belong to the neighbor or the people (Chien et al., 2021b). The barriers are the main problem that are not focused on being resolved and finally resulted in a lack of learning. In this context, Stryja and Satzger (2019) elaborated on the resistance to innovation and its increasing likelihood of insufficient learning capabilities that impacts the decisions. The lack of learning also signifies that the personal abilities are just not resulting in the effective growth of a country but also a barrier to the technology. Moreover, Zhang (2019) discussed the numeric of learning capabilities where the enemies and innovation are faced together and influence the learners. It could be seen with the technological advancement in China that many objectives are more easily achieved than the learners having no ability to grab them. Resistance to innovation is the goal of an organization or people which is required to be achieved otherwise significantly impacts the learning capabilities (Chien et al., 2021a). Additionally, Dejaeghere (2020) analyzed the conceptualization of educational capabilities that are highly influenced by the redressing of inequalities in innovation. It is necessary to understand and be organized for tackling and dealing with the inadvertent situations prevailing in learning abilities. People or countries that are the main resistance to the innovation are considered influential and dominant that are raising the insufficient learning capabilities in China. Finally, Whitfield and Staritz (2021) investigated different traps of learning in the industries that are in deemed need of innovation and sufficient material to eradicate the resistance to technology. When the systems are established for removing or eliminating the resistance to innovation the emergence of learning capabilities takes place. This renders the prevalence of resistance to innovation that is an integral part of insufficient learning capabilities for the people that are learners of a second language.

**H2:** Resistance to innovation significantly influences insufficient learning capabilities.

**Figure 3:**
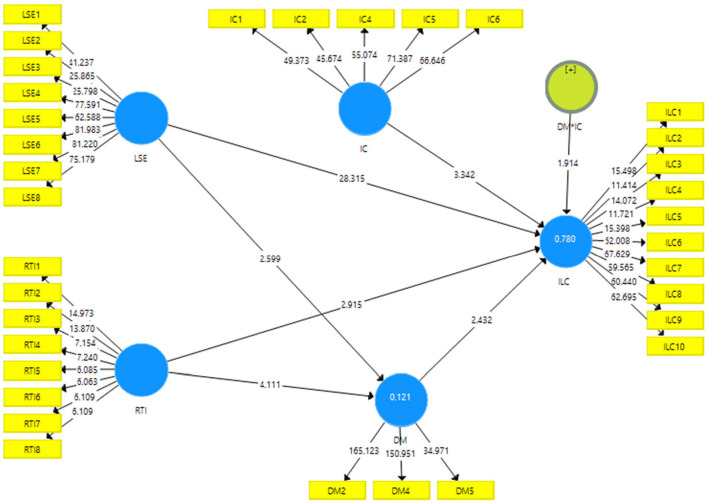
Structural model assessment.

Figure 3 provided the association among variables indicated positive associations among resistance to innovation, lack of self efficacy and insufficient learning capabilities. In addition, demotivation significantly mediates among resistance to innovation, lack of self efficacy and insufficient learning capabilities. Finally, institutional culture significantly moderates among demotivation and insufficient learning capabilities.

### Demotivation as the Mediator

Different countries show the history of people who played a vital role in the destruction and formation of countries like China. In China, the reforms are gradually and increasingly made that indicate the learning abilities and the elements of self-efficacy among people. Thus, Wu et al. (2020) analyzed the demotivation factors and their role in the learning management and lack of self-efficacy. When the learners lack these abilities they are unable to form an organization or could motivate the other learners. The motivation ultimately results in many ways were criminal forces or motivates in a little money or huge amount for the performance of an illegal act. Further, Grenner et al. (2021) investigated the effects and interventions of self-efficacy on the narrative of learning abilities of students and teachers teaching. That act could be upon accomplishment or at the time of revelation could proceed toward punishment but the positive act like motivation toward self-efficacy is a spectacle. Meanwhile, Algraini (2021) assessed human development with education and capabilities perspectives that are associated with the demotivation and lack of self-efficacy. The factors associated with demotivation inserts a negative role in the lack of self-efficacy and insufficient learning capabilities. It is crystal clear that the negative implication always indicates negative results while the positive approach places everlasting impacts on China. Lastly, Pham et al. (2019) examined the relationship between motivated learners and demotivation and its impact on learning abilities. The elements of demotivation when turned into motivation not only uplifts the factors of self-efficacy but also raise the learning capabilities that help the learners of various other languages. These capabilities are helpful in apprising the people to raise their skills of motivation in order to develop in the most probable and efficacious ways.

**H3:** Demotivation significantly and positively mediates the relationship between lack of self-efficacy and insufficient learning capabilities.

**Figure 4:**
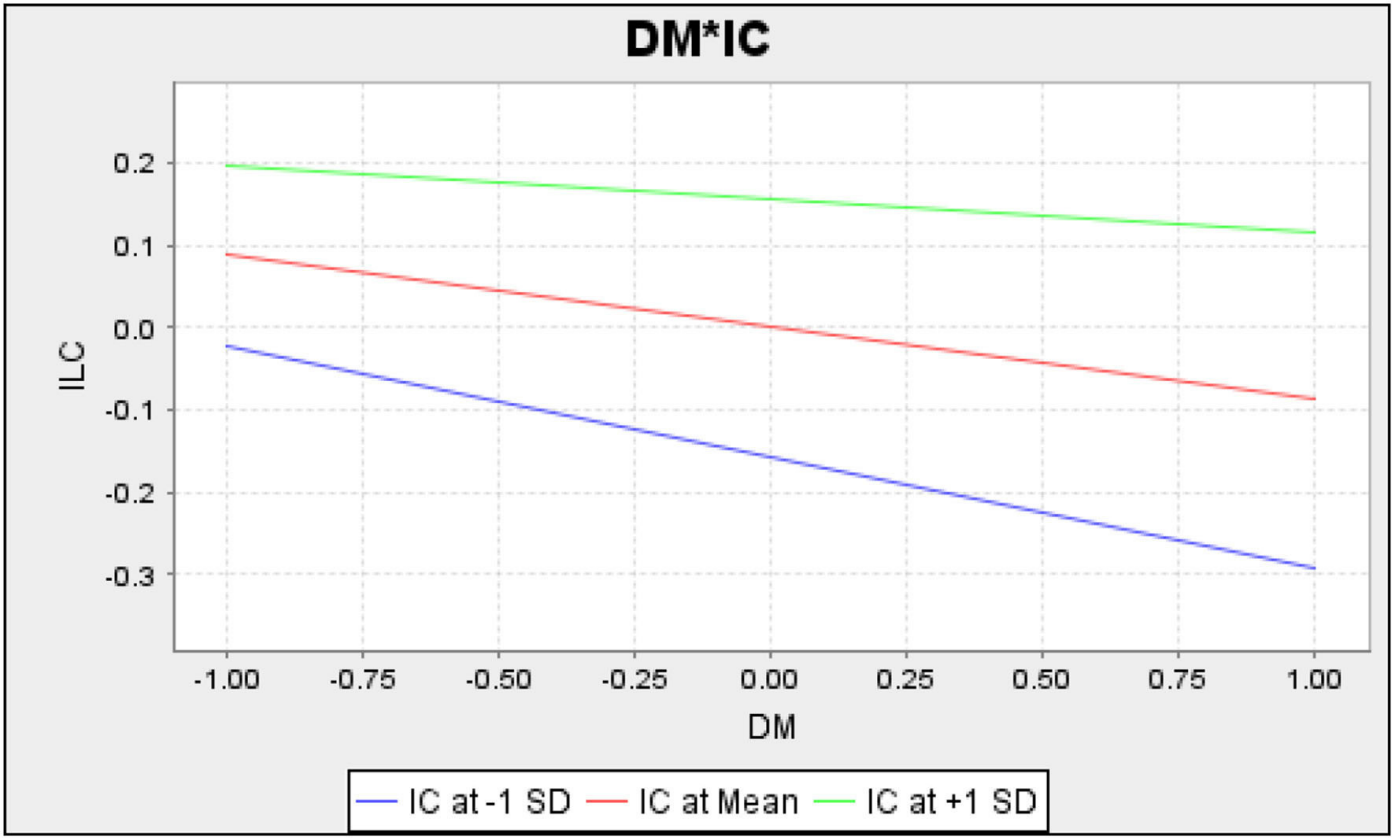
DM*IC.

Figure 4 provided the moderating impact and indicated positive moderating impact of IC among DM and ILC.

All over the world, students are associated with learning abilities and capabilities in various ways with some the motivating as well as demotivating factors. When there is an interference with new technology, the students sometimes force the teachers to disengage them from learning this. Thus, Steiner (2018) discussed the techniques and strategies toward the demotivation that inserts its role over the resistance to innovation and learning. However, the rising standards of innovation have not only improved various fields of businesses but also placed a splendid impact on the field of teaching in China. Teachers are well-acquainted with the techniques and technologies that induced a beneficial impact on their teaching skills. Moreover, Ranci and Arlotti (2019) analyzed the elements linked with resistance to innovation and forcing the systems to not use the policy innovations and their implementations. In fact, some learners after taking participation in the learning faculties are motivated by the usage of innovative techniques. Consequently, Slabbert (2018) applied a capable approach to social work education with the motivation and innovative factors that increase the motivation among learners. The students and different language learners are more convinced and motivated by the technology that is used on different platforms. This usage of innovative techniques among the learners of China helped in eliminating the elements of resistance that prevail in other students. Most of the part of the world where there is a lack of technology also states large demotivation elements. Finally, Rienties et al. (2018) examined the analytics of learning designs and environments that are feasible for the engagement of students to enhance learning. These elements of demotivation significantly elaborate the mediating impact of resistance to innovation and insufficient learning capabilities. The frustrated attitude of people and learners is somehow changed with the consistency of uplifting the motivation elements. These elements not only erased some extent of resisting factors but also improved learning capabilities.

**H4:** Demotivation significantly and positively mediates the relationship between resistance to innovation and insufficient learning capabilities.

### Institutional Culture as the Moderator

Culture poses significance to the environment as well as the facilities for all the learners seeking second language learning. Institutional culture plays a vital role in the learners by imposing the best facilities and best learning skills for the learners. In this context, Adonis and Silinda (2021) analyzed the relationship between transformation and institutional culture in the education sector with the relevance of learning capabilities and demotivation. Learners that are fond of and hold a passion for learning a second language like in China is highly motivated by the attitudes of institutions and learning facilities. Although, a second language is somehow difficult anything could be achieved with motivation and strong aim with strong belief. Further, Bower (2019), explained the motivation and demotivation in language learning with the significant presence of institutional culture. Learning is a necessary element for any task but the institutional cultures are primarily important and play a role in the demotivation and learning capabilities. In China, the systems of education and factors of stereotyping, racism and discrimination are dominant among the second language learners. Additionally, Joshi (2021) discussed the approach of capabilities and human development with the ideology of globalization widely supported by the institutional culture. It is the aim of people and learners to face all the hiding facts belonging to culture but the institutions must form managing places for the learners. These facilities are formed to eradicate the elements which could be highly dangerous for second language learners. Finally, Carey (2018) assessed the influence of process, policy and institutional culture on the decision making to assert the moderating role among learning capabilities. Those elements are also known as main identical elements that pose the environments of demotivation and certainly result in resistance to innovation. The proper establishment of educational systems and the status of students' culture could be motivating for learning capabilities.

**H5:** Institutional culture significantly and positively inserts moderating impact among demotivation and insufficient learning capabilities.

## Methodology

The research examines the role of lack of self-efficacy and resistance to innovation on the insufficient learning capabilities and also examines the mediating role of demotivation among the linkage of lack of self-efficacy, resistance to innovation, and insufficient learning capabilities, and explores the moderating role of institutional culture among demotivation and insufficient learning capabilities. The researchers have applied the questionnaires method to gather the data from selected students. The students of second language learning institutions are the respondents. These students are selected based on simple random sampling. The researchers have distributed the surveys using personal visits to the institutions. The researchers have sent around 495, and after 1 month, only 290 were received, which has an ~58.59% response rate.

**Figure 5:**
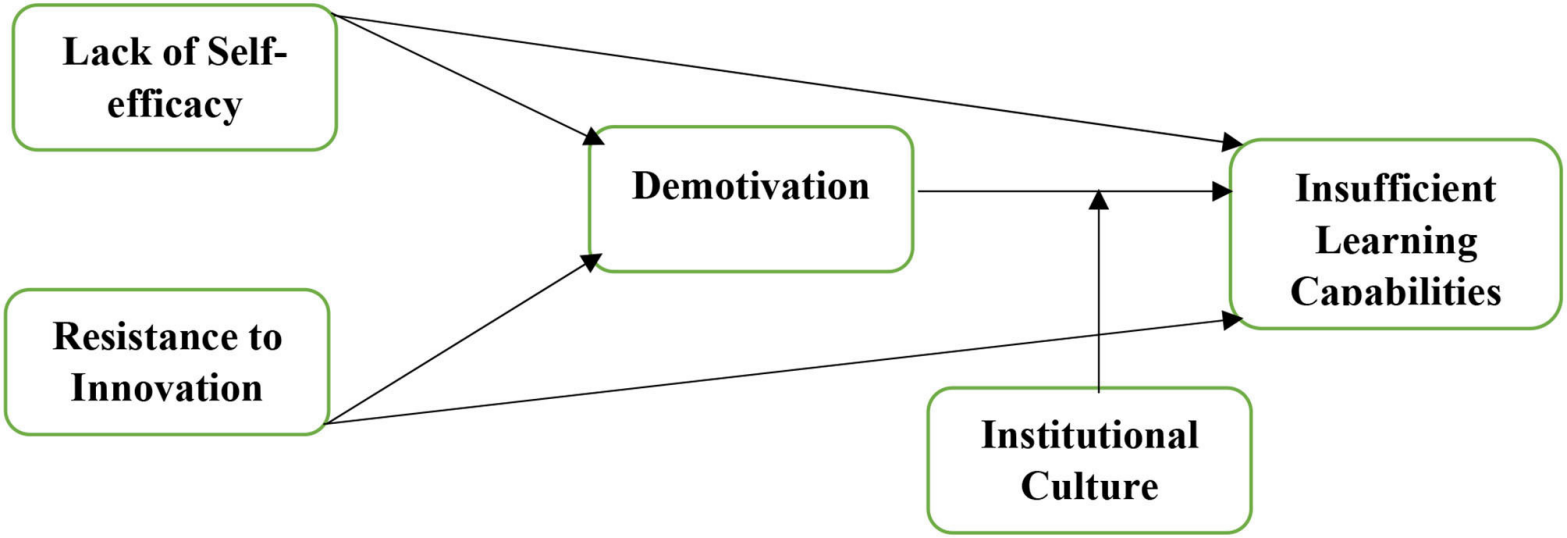
Research model.

The current article has employed the smart-PLS to assess the measurement and structural models. This tool is considered as the best tool to examine the primary data (Hair et al., 2021). In addition, this tool operates effectively even though the researchers have used large data sets and complex frameworks (Hair et al., 2021). The framework of the study is given in Figure 5.

Figure 1 shown above exposed that two predictors have been used such as lack of self-efficacy (LSE) with eight items extracted from the study by De Smul et al. (2018) and resistance to innovation (RTI) with eight items extracted from Hosseini et al. (2016). In addition, demotivation (DM) has been taken as the mediating variable with five items extracted from Çankaya (2018), and institutional culture (IC) has been taken as moderating variable with six items extracted from Naz et al. (2019). Finally, the present research has taken insufficient learning capabilities (ILC) as the dependent variable with ten items and extracted them from Hanson et al. (2021).

## Findings of the Study

Table 1 shows the items' correlation results using factor loadings and average variance extracted (AVE) and also indicates the reliability using Alpha along with composite reliability (CR). The results indicated that loadings and AVE values are >0.50, which exposed a high correlation among items. The values of CR and Alpha are also bigger than 0.70, exposing that the reliability is significant.

**Table 1:** Convergent validity.

**Variables**	**Items**	**Loadings**	**Alpha**	**CR**	**AVE**
Demotivation	DM2	0.973	0.929	0.956	0.878
	DM4	0.974			
	DM5	0.859			
Institutional Culture	IC1	0.867	0.921	0.941	0.761
	IC2	0.863			
	IC4	0.875			
	IC5	0.886			
	IC6	0.870			
Insufficient Learning Capabilities	ILC1	0.643	0.916	0.927	0.565
	ILC10	0.825			
	ILC2	0.593			
	ILC3	0.644			
	ILC4	0.619			
	ILC5	0.666			
	ILC6	0.870			
	ILC7	0.828			
	ILC8	0.871			
	ILC9	0.870			
Lack of self-efficacy	LSE1	0.850	0.942	0.952	0.715
	LSE2	0.771			
	LSE3	0.755			
	LSE4	0.872			
	LSE5	0.859			
	LSE6	0.889			
	LSE7	0.890			
	LSE8	0.866			
Resistance to innovation	RTI1	0.790	0.895	0.895	0.517
	RTI2	0.766			
	RTI3	0.678			
	RTI4	0.684			
	RTI5	0.703			
	RTI6	0.709			
	RTI7	0.705			
	RTI8	0.711			

Table 2 shows the variable correlation results using Fornell Larcker. The results indicated that the column's first value is higher than the rest exposed the association with the variable itself is stronger than the other constructs, and exposed low correlation among variables.

**Table 2:** Fornell larcker.

	**DM**	**IC**	**ILC**	**LSE**	**RTI**
DM	0.937				
IC	0.682	0.872			
ILC	0.232	0.455	0.751		
LSE	0.260	0.436	0.571	0.846	
RTI	0.313	0.381	0.422	0.381	0.719

Table 3 also shows the variable correlation results using cross-loadings. The results indicated that the item values that exposed the association with the variable itself are higher than the rest that exposed the association with the variable itself. They are stronger than the other constructs and expose low correlation among variables.

**Table 3:** Cross-loadings.

	**DM**	**IC**	**ILC**	**LSE**	**RTI**
DM2	**0.973**	0.660	0.223	0.249	0.336
DM4	**0.974**	0.661	0.210	0.234	0.331
DM5	**0.859**	0.595	0.223	0.251	0.199
IC1	0.663	**0.867**	0.390	0.382	0.302
IC2	0.583	**0.863**	0.360	0.349	0.337
IC4	0.634	**0.875**	0.378	0.377	0.353
IC5	0.585	**0.886**	0.411	0.401	0.329
IC6	0.519	**0.870**	0.435	0.388	0.341
ILC1	0.160	0.295	**0.643**	0.392	0.247
ILC10	0.234	0.386	**0.825**	0.862	0.349
ILC2	0.177	0.357	**0.593**	0.390	0.252
ILC3	0.180	0.345	**0.644**	0.422	0.280
ILC4	0.078	0.316	**0.619**	0.383	0.297
ILC5	0.145	0.333	**0.666**	0.463	0.332
ILC6	0.176	0.348	**0.870**	0.785	0.352
ILC7	0.226	0.386	**0.828**	0.863	0.348
ILC8	0.165	0.351	**0.871**	0.780	0.349
ILC9	0.182	0.352	**0.870**	0.792	0.357
LSE1	0.233	0.380	0.727	**0.850**	0.328
LSE2	0.201	0.389	0.661	**0.771**	0.264
LSE3	0.257	0.362	0.615	**0.755**	0.236
LSE4	0.227	0.381	0.762	**0.872**	0.340
LSE5	0.232	0.370	0.743	**0.859**	0.334
LSE6	0.199	0.351	0.770	**0.889**	0.354
LSE7	0.204	0.353	0.775	**0.890**	0.353
LSE8	0.214	0.372	0.818	**0.866**	0.346
RTI1	0.285	0.380	0.497	0.461	**0.790**
RTI2	0.314	0.417	0.490	0.444	**0.766**
RTI3	0.174	0.262	0.187	0.163	**0.678**
RTI4	0.167	0.256	0.184	0.164	**0.684**
RTI5	0.154	0.125	0.107	0.067	**0.703**
RTI6	0.173	0.102	0.112	0.115	**0.709**
RTI7	0.175	0.111	0.111	0.116	**0.705**
RTI8	0.150	0.113	0.115	0.067	**0.711**

*Bold values are larger than the other values in the same row and indicated high correlation with variable itself*.

Table 4 shows the variable correlation results using Heterotrait Monotrait (HTMT) ratio. The results indicated that the values are lower than 0.90 and exposed the association with the variable itself is stronger than the other constructs and exposed low correlation among variables.

**Table 4:** Heterotrait monotrait ratio.

	**DM**	**IC**	**ILC**	**LSE**	**RTI**
DM
IC	0.740				
ILC	0.248	0.498			
LSE	0.281	0.469	0.772		
RTI	0.282	0.321	0.329	0.288	

The results of the direct path revealed that lack of self-efficacy and resistance to innovation has a significant and positive linkage with insufficient learning capabilities and accepts H1 and H2. Table 5 shows the direct linkage among variables.

**Table 5:** Direct path.

**Relationships**	**Beta**	**S.D**.	***T*-statistics**	***p*-values**
DM -> ILC	−0.087	0.036	2.432	0.008
IC -> ILC	0.157	0.047	3.342	0.001
LSE -> DM	0.165	0.063	2.599	0.005
LSE -> ILC	0.804	0.028	28.315	0.000
RTI -> DM	0.250	0.061	4.111	0.000
RTI -> ILC	0.090	0.031	2.915	0.002

The results of the indirect path also exposed that demotivation significantly mediates among lack of self-efficacy, resistance to innovation, and insufficient learning capabilities and acceptance H3 and H4. In addition, the findings also explored that the institutional culture significantly moderates among the linkage between demotivation and insufficient learning capabilities and acceptance H5. Table 6 shows the indirect linkage among variables.

**Table 6:** Indirect path.

**Relationships**	**Beta**	**S.D**.	***T*-statistics**	***p*-values**
DM*IC -> ILC	0.047	0.025	1.914	0.029
RTI -> DM -> ILC	0.022	0.011	2.074	0.020
LSE -> DM -> ILC	0.014	0.008	1.751	0.041

^***^, ^**^, and ^*^ represent significant level at 1%, 5%, and 10%, respectively.

## Discussions and Implications

The results stated that lack of self-efficacy has a positive relation to insufficient learning capabilities. These results are supported by Teng et al. (2018), who show that in second language learning, learning capabilities such as literacy, numeracy, capability to run information and communication technology, critical thinking, creative thinking, personal and social capability, Ethical understanding, and intercultural understanding, are required for effective learning and goals achievement. The development of learning capabilities depends on the perceptions the students have. The lack of self-efficacy in the students forms negative perceptions about learning 2nd language and thus, restricts develop learning capabilities. These results are in line with Sun and Wang (2020), which show that the perception of the students while taking the 2nd language learning classes that they are unable to have the capacity to follow the course, understand the topic, digest the language instructions from tutors, and show their best in performing well during class and test creates hurdles in removing the problems in the way and developing the learning capability. So, the lack of self-efficacy of the students negatively affects learning capabilities development. These results also agree with the study of Zhang and Ardasheva (2019), which confirm that the lack of self-efficacy in the students weakens their potential to do something particular or new, and when students lack self-efficacy, they become unable to develop learning capabilities.

The results also showed that the resistance to innovation adoption has a positive relation to insufficient learning capabilities. These results are also in line with Guan et al. (2020), which posits that for improvement in learning, many skills and abilities are aroused by the adoption of innovation like the use of ICTs, social media, and digital storage devices. The 2^nd^ language learning belongs to a different culture and thus, has a different vocabulary, grammar, and accent. The learning of 2^nd^ language requires critical thinking, capability for ICTs, digital literacy, and social capabilities. In this way, the adoption of innovation assists students in developing learning capabilities. And the language schools which do not care for innovation adoption make it difficult for the students to develop learning capabilities. These results are also in line with McLelland (2018), which posits that the resistance to adopting the innovative learning processes does not allow the students to go ahead to create the dynamic capabilities in them that are essential to learning 2^nd^ language. These results are also supported by Amarakoon et al. (2018), which analyze the impacts of innovation in the form of ICTs on 2^nd^ language learning. The study implies that knowledge, critical thinking, creativity, ethical understanding, and intercultural understanding all are learning capabilities that are linked to innovation ICTs adoption. When students have to face resistance to innovation, it becomes difficult for them to develop learning capabilities.

The results revealed that demotivation is a mediator between lack of self-efficacy and insufficient learning capabilities. These results match with Chen and Zhang (2019), which reveals that when there is a lack of urge in students to learn a 2^nd^ language, they cannot arouse confidence in their own abilities and capacity that they can understand the subject they would be taught, they can complete the curriculum, and eventually succeed to learn the language. The lack of self-confidence does not allow the students to participate in formal and informal learning activities that can develop learning capabilities like analytical thinking, creativity, problem-solving skills, and digital literacy. Hence, the demotivation in students reduces self-efficacy, which further causes insufficient learning capabilities. These results agree with Namaziandost and Çakmak (2020), according to which the lack of self-efficacy causes confusion and doubts in students about the learning processes, training classes, and resources applied; they fail to arouse learning motivation. Because of the lack of motivation, they lack learning capabilities. The results revealed that demotivation is a mediator between resistance to innovation and insufficient learning capabilities. These results match with Turan and Akdag-Cimen (2020), which highlights that the resistance to innovation restrains the institutions from adopting the digital technologies which are used for information and communication purposes. In the modern world, the absence of ICTs keeps the students unaware of learning resources, essentials of the subject, and much other relevant information. The lack of knowledge and information cannot allow the students to bear motivated. Consequently, when the students lack learning motivation, they are not ready for learning how to develop effective learning capabilities.

The results indicated that institutional culture plays a moderating role between demotivation and insufficient learning capabilities. These results are supported by Loon et al. (2020), a study that throws light on the association between institutional culture, demotivation, and insufficient learning capabilities. The article explains that the institutional culture affects demotivation among students and insufficient learning capabilities and their relationships as well. If the institutional culture is unfavorable and discouraging, it cannot arouse learning motivation in students and makes it impossible to develop sufficient capabilities in students. So, in the presence of unfavorable institutional culture, student demotivation leads to insufficient learning capabilities. These results are supported by Saunila (2020), which shows that when the institutional culture is not cooperative and supportive to the teaching faculty, in the 2nd language school system, there is a lack of motivation in the teachers toward their profession. The unfavorable institutional culture causes insufficient learning capabilities. So, the impact of teachers' demotivation on the student's insufficient learning capabilities gets worse.

The present study has both theoretical and empirical implications. The present study contributes to the literature on education. The current study explores the influences of lack of self-efficacy and resistance to innovation on insufficient learning capabilities. In this study, mediating influences of demotivation on the relation from lack of self-efficacy and resistance to innovation to insufficient learning capabilities have been analyzed. It contributes to the literature in the sense that in prior literature, there is no significant study on mediating role of demotivation between lack of self-efficacy, resistance to innovation, and insufficient learning capabilities. In the literature, there has been shown only the direct relation of institutional culture with insufficient learning capabilities but the present study, because addressing the institutional culture as a moderator between demotivation and insufficient learning capabilities, finds a distinctive place in literature. The current study also has empirical significance in all countries where the second language learning has become a dire need in order to contact people belonging to some other culture so that the relations for trading or social development purposes. The learners can have guidance from this study that must overcome the lack of self-efficacy and overcome the resistance to innovation so that they can more efficiently develop learning capabilities. This study guides the institutions, especially those which arrange for 2nd language learning classes, that they must encourage self-efficacy, reduce the resistance to innovation, improve student motivation, and provide institutional support so that insufficient learning capabilities can be reduced.

## Conclusions and Limitations

The objective of the current research was to examine the influences of lack of self-efficacy and resistance to innovation on insufficient learning capabilities. It was to check what role demotivation plays between lack of self-efficacy, resistance to innovation, and insufficient learning capabilities and what institutional culture plays between demotivation and insufficient learning capabilities. A survey was conducted on language schools in China, and information on the relationship between lack of self-efficacy and resistance to innovation, demotivation, institutional culture, and insufficient learning capabilities was collected through questionnaires. The results indicated a positive impact of lack of self-efficacy and resistance to innovation on insufficient learning capabilities. The results showed that students' lack of self-efficacy limits their ability to try anything different, and they are unable to improve their learning abilities when they lack self-efficacy. The results also revealed that many learning capabilities are associated with technological instruments, digital devices, and machines. When students have to face resistance to innovation, they can develop insufficient learning capabilities. The study showed a mediating role of demotivation between lack of self-efficacy, resistance to innovation and insufficient learning capabilities for the lack of self-efficacy and resistance to innovation reduces motivation in students and causes insufficient learning capabilities. The study also concluded that the institutional culture, if it is unfavorable, lowers learning motivation in students and makes them unable to develop learning capabilities. Hence, institutional culture is a moderator between demotivation and insufficient learning capabilities.

Despite the theoretical and empirical significance, the current study also has some limitations and requires more attention from the authors. The study examines only the lack of self-efficacy and resistance to innovation on insufficient learning capabilities. The study does not focus on the human resources management and financial strength of the students and institutions when it determines insufficient learning capabilities. It is the duty of the future scholars also to pay attention to essential factors other than lack of self-efficacy and resistance to innovation for analyzing the insufficient learning capabilities development. The data for checking the relationship between lack of self-efficacy and resistance to innovation, demotivation, institutional culture, and insufficient learning capabilities were acquired from the language schools of China. China has a particular cultural system and education system. So, the study may not be applicable to other education systems and need research on a larger scale to be more general.

## Data Availability Statement

The original contributions presented in the study are included in the article/supplementary material, further inquiries can be directed to the corresponding author.

## Author Contributions

SY confirms being the sole contributor of this work and has approved it for publication.

## Conflict of Interest

The author declares that the research was conducted in the absence of any commercial or financial relationships that could be construed as a potential conflict of interest.

## Publisher's Note

All claims expressed in this article are solely those of the authors and do not necessarily represent those of their affiliated organizations, or those of the publisher, the editors and the reviewers. Any product that may be evaluated in this article, or claim that may be made by its manufacturer, is not guaranteed or endorsed by the publisher.
